# Validation of Omron HBP-1300 professional blood pressure monitor based on auscultation in children and adults

**DOI:** 10.1186/s12872-015-0177-z

**Published:** 2016-01-13

**Authors:** Linghui Meng, Di Zhao, Yan Pan, Wenqing Ding, Qing Wei, Hua Li, Pingjin Gao, Jie Mi

**Affiliations:** Department of Epidemiology, Capital Institute of Pediatrics, 2 Yabao Road, Beijing, 100020 China; Shanghai Key Lab of Hypertension, Shanghai Institute of Hypertension, Shanghai, 200025 China

**Keywords:** Validation study, Electronics, Sphygmomanometer

## Abstract

**Backgroud:**

To determine whether the professional Omron HBP-1300 blood pressure (BP) monitor meets American Association for the Advancement of Medical Instrumentation (AAMI) accuracy standards in Chinese children and adults.

**Method:**

According to the AAMI protocol, simultaneous auscultatory measurements by two observers using a mercury manometer were obtained in participants using the Omron HBP-1300. Triple measurements were obtained after a minimum 5-min rest with a 1-min interval between adjacent measurements.

**Results:**

A total of 85 participants submitted to 255 doctor-measured BP and 255 successful professional monitor readings. The initial auscultation systolic BP was <100 mmHg in 25 participants (29.4 %), 100–160 mmHg in 53 participants (62.4 %), and >160 mmHg in seven participants (8.2 %). All of the simultaneous measurements agreed to within ±10 mmHg, while 95 % agreed to within ±4 mmHg for both systolic and diastolic BP, and the consistency between two observers was satisfactory. The difference between the devices was -1.3 ± 3.6 mmHg for systolic BP and 0.7 ± 3.8 mmHg for diastolic BP and by AAMI method 1, which met this guideline. The average difference between two devices by AAMI method 2 was 1.4 ± 3.2 mmHg for systolic BP and 1.0 ± 3.9 mmHg for diastolic BP, which met this guideline.

**Conclusion:**

The professional BP monitor Omron HBP-1300 is desirable for measuring the BP for Chinese children and adults.

**Electronic supplementary material:**

The online version of this article (doi:10.1186/s12872-015-0177-z) contains supplementary material, which is available to authorized users.

## Background

Many hypertension survey protocols use automatic digital devices for measurements since they have no observer error and require no strict training. Clinicians are increasingly developing confidence that automated blood pressure (BP) device readings are comparable to those of auscultatory manual devices [1, 2]. Meanwhile, trial evidence indicates that even small differences of 2–4 mmHg in systolic BP (SBP) are clinically important, thus accurate measurements are vital. A BP-measuring device that can pass the American Association of Medical Instrumentation (AAMI) standard testing [3] with reliable accuracy would be a desirable one for either clinic or research. The professional blood pressure monitor Omron HBP-1300 might potentially be a new monitor meeting that need.

## Methods

### Introduction of the AAMI standard

The standard advises testing over a range of arm sizes and BP. Subjects should undergo a variety of BP readings to determine whether the device is accurate for all BP ranges. All device cuff sizes to be marketed should be studied.

The AAMI standard requires to include data of all participants who entered into the validation study, an explanation of the technical problems preventing inclusion of the required data, and a full explanation of and the reasons for data exclusion. For inter-observer comparisons, the AAMI standard states that all of simultaneous measurements should vary within ±10 mmHg and ≥90 % of those measurements should vary within ±5 mmHg between different observers for both SBP and DBP. Once the measurement meets these criteria, the average of these measurements is used for the analysis.

The AAMI standard provides two methods for accuracy determination on automated sphygmomanometers in which the auscultatory method is used as a reference standard. Method 1 requires a minimum of 255 observations from ≥85 participants. If a participant contributes fewer than three data sets (paired observer and device measurements), additional participants are tested to reach a minimum of 255 observations. The AAMI standard requires that the variability of the automated device for both SBP and DBP should be within 4 (mean) ± 8 (SD) mmHg. Method 2 was developed because of concerning that method 1 might qualify a device with clinically unacceptable error in terms of allowing the upper limit of both the mean difference and the SD. Method 2 uses the average of three measurements for each participant to replicate the use of multiple measurements to establish a diagnosis of hypertension [4, 5].

### Human subjects

The Chinese Capital Institutes of Pediatrics completed the collection of data for 35 children and merged them with the data of 50 adults and adolescents provided by the Shanghai Institute of Hypertension. Adult subjects were mostly outpatients and inpatients of Ruijin Hospital. The pediatric and adolescent subjects were mostly recruited from routine checkups performed in the school or community. Participants with known significant heart rhythm disorders were excluded.

### Human observers

All observers were pediatricians or doctors whose accuracy and reliability in BP measurement in children and adults were verified through strict training and passing of a strict inspection.

### BP measurements

Pairs of observers were trained to perform auscultatory measurements by reporting of the first and fifth Korotkoff sounds as SBP and DBP in adults and the first and fourth Korotkoff sounds as SBP and DBP in children < 12 years old. The testing was performed in a warm and quiet room. Each participant was seated with the back supported, legs uncrossed, and feet on the floor. The observers measured the circumference at the midpoint of the arm and selected an appropriate cuff, the width of which was as close as possible to 40 % of the arm circumference. The cuff tubing had a three-way stopcock inserted to alternate the manual auscultatory readings. After each subject rested for at least 15 min, BP was measured on the left upper arm at the level of the heart and the lower arm passively supported. The interfacing two measurements were separated by at least 1 min intervals. Heart rate was recorded in beats/min at each visit. Then the button of start was pressed and the measurements started automatically [6].

The cuffs were applied to the left upper arm with the cuff artery marker positioned over the point of brachial artery palpation. The observers used a calibrated aneroid sphygmomanometer (Diving Fish upper arm type mercury desktop device; Jiangsu Diving Fish Medical Equipment Co. LTD.). The test devices underwent identical calibration procedures. The stethoscope had dual earpieces to allow simultaneous auscultation by both trained observers. The observers were blinded from each other and from the device during the BP measurements. If the readings from different observers varied >4 mmHg, the measurements was repeated until the variability was less than 4 mmHg. Studies were stopped or rejected if subjects had arrhythmia, could not sit still for the study. Studies were also excluded if the average systolic readings from different measurements differed by >10 mmHg or the diastolic reading varied by >5 mmHg after several attempts as required in the protocol.

### Protocol

The protocol defined in ANSI/AAMI/ISO81060-2:2013 requirement [7] was used in this study. An initial and stabilization manual BP measurement was performed for BP classification. Subsequent automated manual readings were alternated with at least 1 min separating consecutive measurements. Each participant underwent at least three pairs of automated vs. manual readings. The variability of SBP or DBP values between two observers should be ≤5 mmHg. The two observers’ readings for each measurement were averaged before comparison with the corresponding paired measurement by the automated device. The device should be documented to have passed the ANSI/AAMI/ISO81060-2:2013 requirement through the data analysis.

### Test devices

The HBP-1300 Professional Blood Pressure Monitor is easy to use and has a durable design that includes a built-in handle that makes it easily portable. It is designed for use in professional settings and is clinically proven to produce fast and reliable results. The monitor is compatible with a series of wipe-clean GS cuffs ranging from SS (12–18 cm) to XL (42–50 cm). It comes with an AC adapter as well as a rechargeable battery pack. The device is fitted with a shock-proof bumper that protects it from accidental damage and comes with a fully automatic oscillometric and manual auscultatory mode. It is possible to manually adjust the inflation pressure to improve patient comfort.

### Statistics

The data were recorded on paper and subsequently transferred into a database built using Epidata software. The statistics were calculated by SPSS 20.0 and Medcalc software. Bland-Altman plots were constructed with average observer SBP or DBP values on the x-axis and Spot error values on the y-axis, and the figures show the ±1.96 SD limits.

### Ethics statement

Written informed consent was obtained from each participant and/or their parents or guardians. The study protocol was approved by the Institutional Review Board and Ethics Committee of Capital Institute of Pediatrics, China.

## Results

A total of 85 participants (49 males, 58 %) were recruited into the study, and there were 255 valid paired comparisons. No data points were eliminated during the analyses; hence, there were 255 data sets. The average age was 25.9 ± 15.6 years. The characteristics of the participants are shown in Table 1. Dataset is seen in Additional file 1.

**Table 1:** Characteristics of the study population

	Mean ± SD	Range
Age (years)	25.9 ± 15.6	4-72
Male/female	49:36:00	-
Pulse (beats/minute)	82 ± 15	51-120
Arm circumference (cm)	25.8 ± 8.3	15.7-47
Systolic blood pressure(mmHg)	119 ± 28	84-210
Diastolic blood pressure(mmHg)	72 ± 18	42-120

There were 5 size cuffs provided to Omron HBP-1300 Professional Blood Pressure Monitor in the market. The cuff range, cuff size labels, and the numbers of children and adults in each category were presented in Table 2.

**Table 2:** Cuff sizes and numbers in each size group

Cuff range (cm)	Cuff Size label	Need 85/^2^n(numbers of Cuff sizes) or 85/10=9	Numbers in this study
R 12-18	SS	9	22
R 17-22	S	9	13
M 22-32	M	9	25
L 32-42	L	9	18
XL 42-50	XL	9	9

The initial auscultation SBP was <100 mmHg in 25 participants (29.4 %), 100–160 mmHg in 53 participants (62.4 %), and >160 mmHg in seven participants (8.2 %). The initial DBP was <60 mmHg in 23 participants (27.1 %), >100 in nine participants (10.6 %), and ≥85 mmHg in 53 participants (62.4 %). The arm circumference was <25 cm for 46 participants, 25–30 cm for 25 participants, and >35 cm for 12 participants. There were twice as many patients as the number required for minimal compliance with the AAMI protocol.

### Error statistics

The spot device mean SBP error was -1.3 ± 3.6 mmHg: 84.7 % of the error readings were within ±4 mmHg, 98 % were within ±5 mmHg, and 100 % were within ±10 mmHg. All SBP values passed AAMI requirements. For DBP, the mean error was 0.7 ± 3.8 mmHg: 85.9 % of the DBP errors were within ±4 mmHg, 98.4 % were within ±10 mmHg, and 100 % were within ±15 mmHg. All the values exceeded the AAMI minimum requirements (passed method 1). The average difference between observers and the device by the AAMI method 2 was 1.4 ± 3.2 mmHg for SBP and 1.0 ± 3.9 mmHg for DBP, which met this guideline.

### Bland-Altman plots

**Fig. 1:**
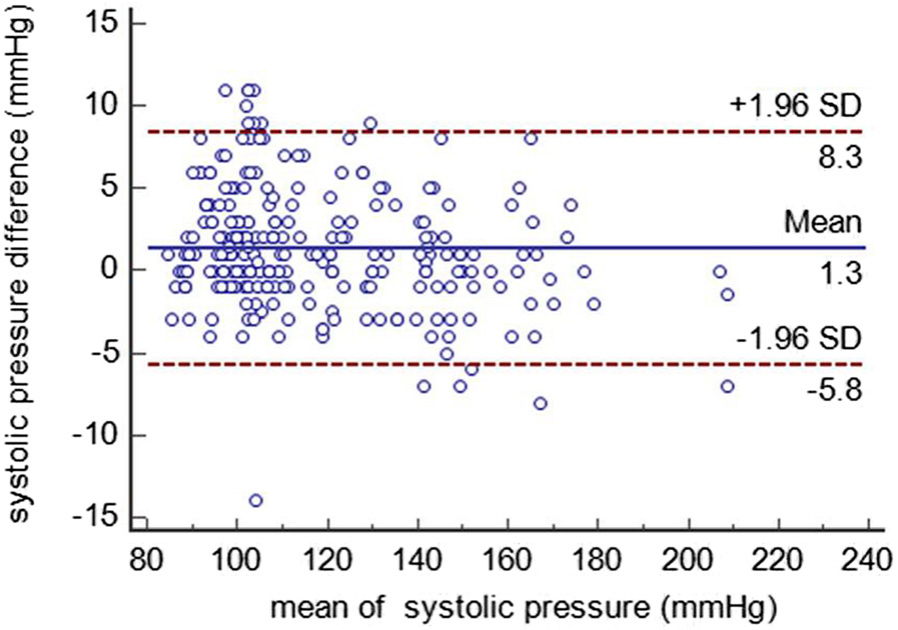
Agreement between the test and reference methods for systolic pressure. The X axis in Fig. 1 represents the mean of systolic blood pressure of the HBP-1300 Professional Blood Pressure Monitor and the sphygmomanometer; and the Y axis represents the difference between systolic blood pressure measured by HBP-1300 Professional Blood Pressure Monitor and the systolic blood pressure measured by the sphygmomanometer

**Fig. 2:**
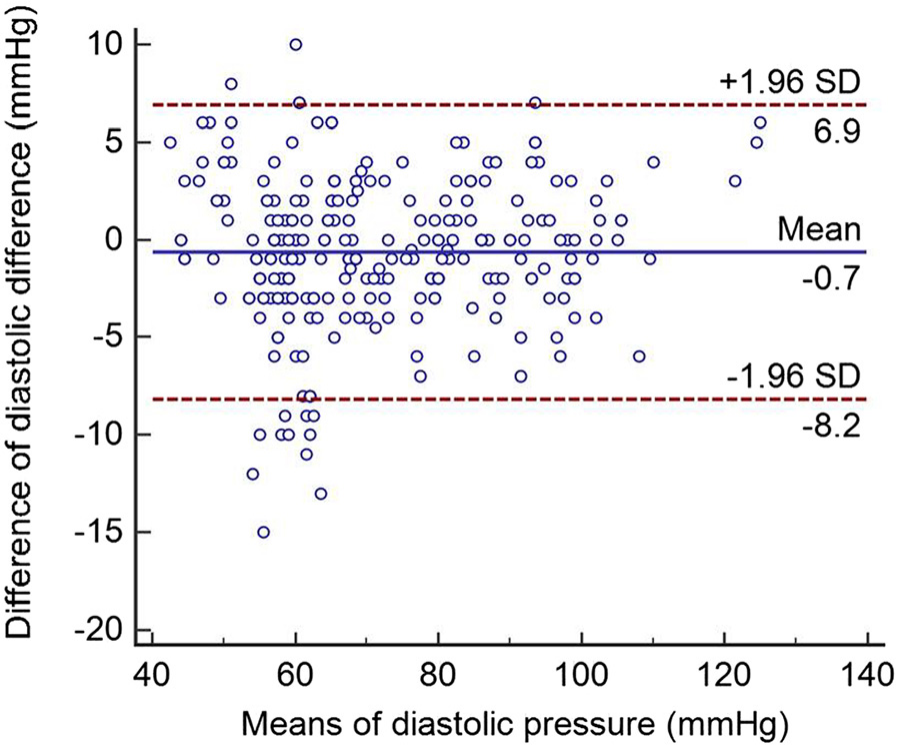
Agreement between the test and reference methods for diastolic pressure. The X axis in Fig. 2 represents the mean of diastolic blood pressure of the HBP-1300 Professional Blood Pressure Monitor and the sphygmomanometer; and the Y axis represents the difference between diastolic blood pressure measured by HBP-1300 Professional Blood Pressure Monitor and the diastolic blood pressure measured by the sphygmomanometer

Bland-Altman plots were constructed with average observed SBP or DBP values on the x-axis and spot error values on the y-axis (Figs. 1 and 2). The figures show ±1.96 SD limits. No pattern of BP underestimation or overestimation is discernable from the data distribution.

## Discussion

The professional HBP-1300 device was tested against a population of adults and children to improve researchers’ and clinicians’ confidence that the device would give excellent results in an epidemic survey and in clinics in China. In the current study, the device met or exceeded the testing requirements of the AAMI American National Standard.

Since hypertension is a chronic disease and BP is affected by various factors [8], accurate BP measurements are critical to its proper treatment. Virtually all digital BP monitors sold within the past decade use the oscillometric technique to measure BP. The replacement of mercury sphygmomanometers with electronic sphygmomanometers has become common [9]. A device that can pass the AAMI standard testing [3] with reliable accuracy would be desirable in the research and clinical settings.

The HBP-1300 has built-in auscultatory and oscillometric functionality for measuring BP. In the current study, the oscillometric model was tested according to the AAMI protocol. The auscultatory model reflects the habits of clinical physicians in the clinical setting. In the end, the average difference between the two doctors was very low. The Bland-Altman plots showed narrow differences between the observers and the device, and no important differences were observed over the measured BP ranges.

In many parts of the world, healthcare facilities and regulatory bodies require testing according to the British Hypertension Society (BHS) protocol [10]. The BHS protocol is not a standard like the AAMI document. The BHS data analyses result in grades of A, B, C, or D depending on the error percentages within ±5, ±10, and ±15 mmHg compared with the observer data. To achieve the highest grade, A, on the BHS protocol data analysis, at least 60, 85, and 95 % of the error readings must be within 5, 10, and 15 mmHg, respectively, from each chosen observer value. The spot device mean SBP error was -1.3 ± 3.6 mmHg: 84.7 % of the error readings were within ±4 mmHg, 98 % were within ±5 mmHg, and 100 % were within ±10 mmHg. All of the SBP values passed AAMI requirements. For DBP, the mean error was 0.7 ± 3.8 mmHg: 85.9 % of the DBP errors were within ±4 mmHg, 98.4 % within ±10 mmHg, and 100 % were within ±15 mmHg. All of the values exceeded the AAMI minimum requirements (passed method 1). The tested device should be useful in various sites of medical-care delivery because of its accuracy.

According to ISO 81060-2: 2013 the Criterion1 uses the 255 individual test - REFERRENCE differences to determine the performance of the SPHYGMOMANOMETER – UNDER - TEST. As a result, the calculated standard deviation, *s*_*n*_ (or precision) will reflect both intra-subject and inter-subject variability. However, the allowable standard deviation is constant even when the mean of difference, mean of _*n*_ (or bias) is large. Criterion uses the average of the differences from each subject, so the calculated sm reflects only inter subject variability, and a large intra – subject variability can still pass this method. Criterion 2 attempts to prevent that by reducing the allowable sn as _*n*_ increases, _(_*s*_*n*_ = 8.00mmHg versus *s*_*m*_ = 6.95mmHg_)_, thus addressing both bias and inter-subject precision. In this manuscript both the criterions were used to test the validation of this sphygmomanometer and it has passed the request of both the criterions successfully.

## Conclusion

The professional BP monitor Omron HBP-1300 is desirable for measuring the BP for Chinese children and adults.

## Availability of supporting data

The database has been uploaded as a supplementary material file on the website.

## Additional file

Additional file 1:
**Validation of** **OMRON-1300** **database.** 85 subjects and 51 variables were included in the database, ‘NO’ represented the identify number, ‘A’ represented the 1^st^ examiner, and ‘B’ represented the 2^nd^ examiner, ‘SBP’ was systolic blood pressure, ‘DBP’ was diastolic blood pressure, ‘1’ to ‘7’ represented the times of measurements, ‘HR’ was heart rates per minute, ‘SEX’ was the gender, ‘AGE’ was the age of the subjects. (XLSX 24 kb)
